# The effect of alienation on depression among Chinese caregivers of children with autism: family resilience as a moderator

**DOI:** 10.3389/fpsyt.2026.1760780

**Published:** 2026-03-13

**Authors:** Lin Zheng, Xin Liu

**Affiliations:** 1 Department of Sociology, School of Humanities and Social Sciences, Yanbian University, Yanji, China; 2 Office of Academic Affairs, Meizhouwan Vocational Technology College, Putian, Fujian, China

**Keywords:** alienation, autism, caregiver, depression, family resilience

## Abstract

**Background:**

Caregivers of children with autism spectrum disorder (ASD) in China often encounter stigma and social isolation, which may heighten alienation and increase depression risk. Family resilience—the strengths and resources within the family system, may help protect caregiver mental health. This study examined the association between alienation and depression among Chinese caregivers of children with ASD and tested whether family resilience moderates this relationship.

**Methods:**

A cross-sectional survey of 205 caregivers in Jilin Province assessed alienation, depressive symptoms, and family resilience using validated Chinese instruments. Exploratory factor analysis was used to confirm the structure of the Family Resilience (FaRE) Questionnaire for this population. Hierarchical regression analyses examined the association between alienation and depression and the moderating effects of family resilience subdimensions, controlling caregiver demographics.

**Results:**

Alienation was positively associated with depression (β = 0.61, p < 0.001). Three dimensions of family resilience—communication/cohesion, perceived social support, and faith/spiritual beliefs—significantly weakened this association (interaction p < 0.05). The family coping dimension did not show a moderating effect.

**Conclusion:**

Alienation is a significant risk factor for depression among Chinese caregivers of children with ASD, but specific resilience processes within families can buffer this impact. Interventions aimed at reducing caregiver alienation and strengthening communication, support networks, and shared belief systems may help mitigate depression in this population.

## 1 Introduction

Autism spectrum disorder (ASD) has shown a steady rise worldwide. In the United States, recent surveillance data indicate that about 1 in 36 eight-year-old children meets diagnostic criteria for ASD, reflecting a continued upward trend (1). China has reported similar increases; contemporary reviews estimate that approximately 0.26–1% of the population may be individuals with ASD, and newer meta-analyses suggest that prevalence among Chinese children approaches international levels (2, 3). As diagnoses rise, increasing numbers of families must undertake long-term caregiving, elevating concerns regarding the well-being of caregivers of children with ASD.

Terminology note: We use person-first language (e.g., “children with ASD”) throughout to emphasize individuals rather than diagnoses, consistent with bias-free and inclusive language guidance for scholarly writing (4). We acknowledge that preferences vary and that some autistic individuals and communities prefer identity-first language; future work should follow participants’ self-identified terminology where feasible.

From a diversity and equity perspective, caregivers of children with ASD in China remain under-represented in the international literature and may face structural barriers such as stigma and constraints in access to supports and services (5, 6). These inequities may place additional strain on families with limited resources or restricted access to formal services, increasing risks of social exclusion and poor mental health. Accordingly, examining alienation and resilience among Chinese caregivers can inform equity-oriented strategies to address inequities in caregiver mental health (7).

Against this backdrop of structural barriers and uneven support, caregivers of children with ASD frequently experience considerable psychological distress. Recent studies in China and abroad show high rates of stress, anxiety, and depression among these caregivers (8–15). Beyond intensive daily caregiving demands, many caregivers confront persistent stigma rooted in misconceptions about ASD (16, 17). Such stigma often takes the form of blame or disapproval, contributing to experiences of affiliate stigma or social exclusion (5, 18–23). Repeated exposure to stigmatizing interactions can foster social withdrawal and a sense of alienation, characterized by perceived disconnection from society and interpersonal relationships. This alienation may compound the emotional burden of caregiving and heighten vulnerability to deteriorating mental health.

A growing body of research links alienation and related constructs—such as internalized stigma, perceived discrimination, and chronic loneliness—to adverse psychological outcomes. Evidence from recent Chinese studies indicates that feelings of alienation are associated with elevated depressive symptoms even after accounting for other stressors (24). Among caregivers of individuals with developmental disabilities, stigma-related alienation similarly predicts poorer well-being and increased depression (25). Given that depressive symptoms affect an estimated 40–50% of caregivers of children with ASD worldwide (26), understanding how alienation contributes to depression is an urgent research priority.

Not all caregivers exposed to adversity experience poor mental health. Many demonstrate resilience when navigating the challenges of raising children with ASD. Family resilience refers to the processes through which families withstand, adapt to, and recover from stress (27). Conceptually, different dimensions of family resilience may buffer the psychological impact of alienation through distinct mechanisms. For example, communication and cohesion within the family may reduce the emotional consequences of alienation by fostering emotional connectedness and shared understanding, whereas perceived social support can counteract social withdrawal by extending supportive resources beyond the immediate family. Faith or spiritual beliefs may further promote meaning-making and optimism, helping caregivers cope with marginalizing experiences. Prior research has consistently shown that higher levels of family resilience are associated with better psychological adjustment among caregivers of children with ASD (28, 29).

This study examines whether family resilience mitigates the impact of alienation on depression among caregivers of children with ASD in China. We hypothesized that alienation would be positively associated with depressive symptoms (H1) and that specific dimensions of family resilience would moderate this association by weakening the relationship between alienation and depression (H2). Clarifying these relationships can deepen understanding of both risk and protective processes and inform interventions aimed at reducing caregiver isolation and strengthening family resilience in educational and community contexts.

## 2 Methods

### 2.1 Participants and procedure

The participants were primary caregivers of children with ASD recruited from community organizations, rehabilitation centers, and parent support groups in Jilin Province, Northeast China. From a diversity and equity perspective, access to ASD-related services and supports may vary across settings, and caregivers may rely on different combinations of formal and informal resources. This recruitment strategy therefore captures caregivers navigating varying levels of service accessibility and social inclusion. This design helps contextualize alienation as an equity-relevant psychosocial experience linked to differential opportunities for support and participation. Between June and August 2023, a total of 205 caregivers aged 22–76 years completed structured questionnaires. Researchers collaborated with local administrators to identify and invite eligible participants.

Eligible participants were primary caregivers (parents or grandparents) of a child formally diagnosed with ASD who had at least three months of continuous caregiving experience. This criterion was used to ensure that caregivers had sufficient exposure to daily caregiving demands and had moved beyond the initial adjustment period, allowing for more stable assessments of psychosocial experiences. To further ensure inclusion of caregivers actively involved in daily care, those not currently co-residing with their child were excluded. Each family contributed one caregiver, defined as the individual primarily responsible for the child’s daily care.

Among the respondents, 80.5% were female caregivers, 19.5% were male caregivers. Most caregivers were middle-aged, with approximately 39% in their 30s and 29% in their 40s. Educational attainment was moderate: 48.8% had junior high school education or below, while 51.2% had at least a high school education. Nearly 43% reported one or more chronic health conditions.

Participants completed the survey either on paper or online after providing written informed consent. The survey required approximately 10–15 minutes to complete, and researcher assistance was available if needed. The study protocol was approved by the Institutional Review Board of Jeonbuk National University, South Korea (IRB2023-06-003-002), as part of the author’s doctoral research project at Jeonbuk National University, where the study was initiated and overseen during the author’s PhD training. Data were collected in China through collaborating community organizations and service agencies with administrative permission from site leaders. The present manuscript reports a secondary analysis of this previously collected dataset; no additional recruitment or data collection was conducted for the current study. Participation was voluntary and anonymous, written informed consent was obtained, and no identifying information was collected. Questionnaires were considered valid if they (a) contained no substantial missing data on key study variables (alienation, depression, and family resilience) and core covariates, and (b) showed no obvious internal inconsistencies in basic demographic information. Based on these criteria, 205 of the 220 returned questionnaires were retained for analysis.

Given the exploration nature of the moderation analyses, the sample size (N = 205) is comparable to that used in prior studies examining caregiver psychological outcomes using hierarchical regression approaches. Although no formal *a priori* or *post hoc* power analysis was conducted, the sample size was considered adequate for exploratory modeling with multiple covariates, and the findings should be interpreted with appropriate caution.

### 2.2 Measures

#### 2.2.1 Depression

Caregiver depressive symptoms were assessed using the 20-item Center for Epidemiologic Studies Depression Scale (CES-D), Chinese version. Items measure the frequency of depressive symptoms (e.g., sadness, hopelessness, sleep disturbance) experienced during the past week on a 4-point scale ranging from 0 (“rarely or none of the time”) to 3 (“most or all of the time”). Higher scores indicate greater depressive symptoms.

Although the CES-D is commonly reported using a summed total score (range=0–60) and associated cutoff values in prior research, all analyses in the present study were conducted using mean item scores. This approach was adopted to ensure consistency across study measures and to facilitate interpretation in regression and moderation analyses. The Chinese version of the CES-D has demonstrated strong psychometric properties in prior studies (30; 31). In the present sample, internal consistency was excellent (Cronbach’s α = 0.95).

#### 2.2.2 Alienation

Caregivers’ sense of alienation was measured using a revised Chinese version of the Generalized Social Alienation Scale (GSAS), adapted by Yang (32) based on Jessor’s conceptualization of alienation (33). The scale assesses perceived social disconnection, isolation, and powerlessness (e.g., “I feel like I don’t really belong in society”).

Items were rated on a 4-point Likert scale ranging from 1 (“strongly disagree”) to 4 (“strongly agree”), with higher scores indicating greater alienation. Mean item scores were used in all analyses. The revised adult version has demonstrated good psychometric properties in prior research on social estrangement and perceived discrimination (34). In the present study, the scale showed excellent internal consistency (Cronbach’s α = 0.94).

#### 2.2.3 Family resilience

Family resilience was assessed using the Family Resilience Questionnaire (FaRE), originally developed by Faccio et al. (28) and translated and psychometrically validated in a Chinese sample (8, 35). The scale is grounded in a systemic resilience framework and assesses four theoretically distinct dimensions: (a) communication and cohesion, reflecting emotional connectedness and open family communication; (b) perceived social support, capturing both internal and external support resources; (c) family coping ability, indicating perceived competence in managing stress and adversity; and (d) faith/spiritual beliefs, reflecting shared belief systems and meaning-making processes. All items were rated on a 4-point Likert scale (1 = strongly disagree; 4 = strongly agree), with higher scores indicating greater family resilience.

Although a Chinese version has been validated (8), it has not been previously examined among caregivers of children with ASD; therefore, we conducted an exploratory factor analysis (EFA) to evaluate its factorial structure in the present sample. Bartlett’s test of sphericity was significant (χ² = 3948.81, p < 0.001), and the Kaiser–Meyer–Olkin (KMO) measure was 0.953, indicating excellent sampling adequacy. Principal axis factoring with varimax rotation was employed. Factors were retained based on eigenvalues greater than 1.0 and inspection of the scree plot.

Five items (Items 1, 8, 9, 10, and 17) were removed due to low factor loadings (< 0.63) or conceptual overlap. All retained items loaded strongly (≥ 0.63) on their intended factors and showed no substantial cross-loadings. The final 19-item solution preserved the original four-factor structure proposed by Faccio et al. The four subscales demonstrated strong internal consistency (Cronbach’s α = 0.88–0.92), and the overall scale showed excellent reliability (α = 0.96). The KMO and Bartlett’s test results are reported in **Table 1**.

**Table 1:** Kaiser–Meyer–Olkin (KMO) and Bartlett’s test of sphericity for the family resilience scale.

Test		Value	Criterion
Kaiser–Meyer–Olkin (KMO) Measure of Sampling Adequacy		0.953	≥ 0.60
Bartlett’s Test of Sphericity	Approx. Chi-square	3948.813	—
	df	190	—
	p-value	<.001	<.05

#### 2.2.4 Covariates

Several caregiver sociodemographic characteristics were included as covariates: gender (0 = male, 1 = female), age (years), education level (0 = junior high school or below, 1 = high school or above), and presence of a chronic health condition (0 = no, 1 = yes). These variables have been consistently associated with caregiver mental health outcomes in ASD research (29, 36) and were included to adjust for their potential confounding effects.

### 2.3 Data analysis

We first conducted descriptive analyses to summarize caregiver characteristics and the distributions of the main study variables. Independent t-tests and one-way ANOVAs were used to examine group differences in depression, alienation, and the family resilience subdimensions across key demographic categories (e.g., gender, education). Pearson correlations were then calculated among alienation, depression, and the resilience subdimensions to examine bivariate associations and assess potential multicollinearity. For descriptive purposes, an overall family resilience score was included in the correlation analyses to provide a general overview of bivariate associations. However, in the regression and moderation analyses, only the four resilience subdimensions were examined to avoid redundancy and multicollinearity and to maintain a clear focus on distinct family resilience processes.

To test the primary hypotheses, we estimated a series of hierarchical linear regression models. Depression was specified as the dependent variable and alienation as the main independent variable. Family resilience was examined as a moderator through separate moderated regression models for each of the four subdimensions (communication/cohesion, perceived social support, family coping ability, and faith/spiritual beliefs). No model was estimated for an overall resilience score, as the analytic focus was on the distinct dimensions.

Each regression analysis followed a three-model procedure. Model 1 included the control variables (caregiver gender, age, education, and chronic illness). Model 2 added the mean-centered main effects of alienation and the relevant resilience subdimension. Model 3 included the interaction term (Alienation × Resilience Subdimension). Mean-centering was used to reduce multicollinearity. Moderation effects were evaluated based on the significance of the interaction term and the change in R² from Model 2 to Model 3 (37).

**Figure 1:**
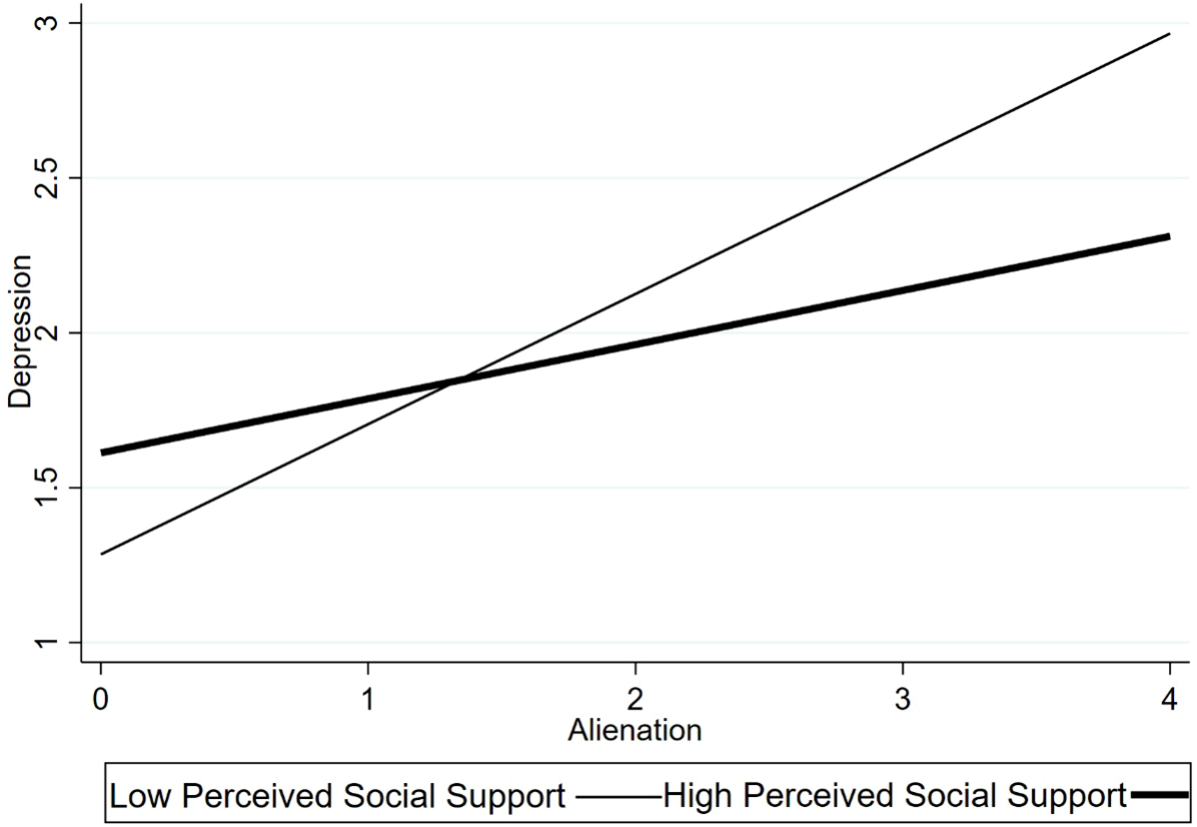
Interaction between alienation and family communication/cohesion in predicting depression.

**Figure 2:**
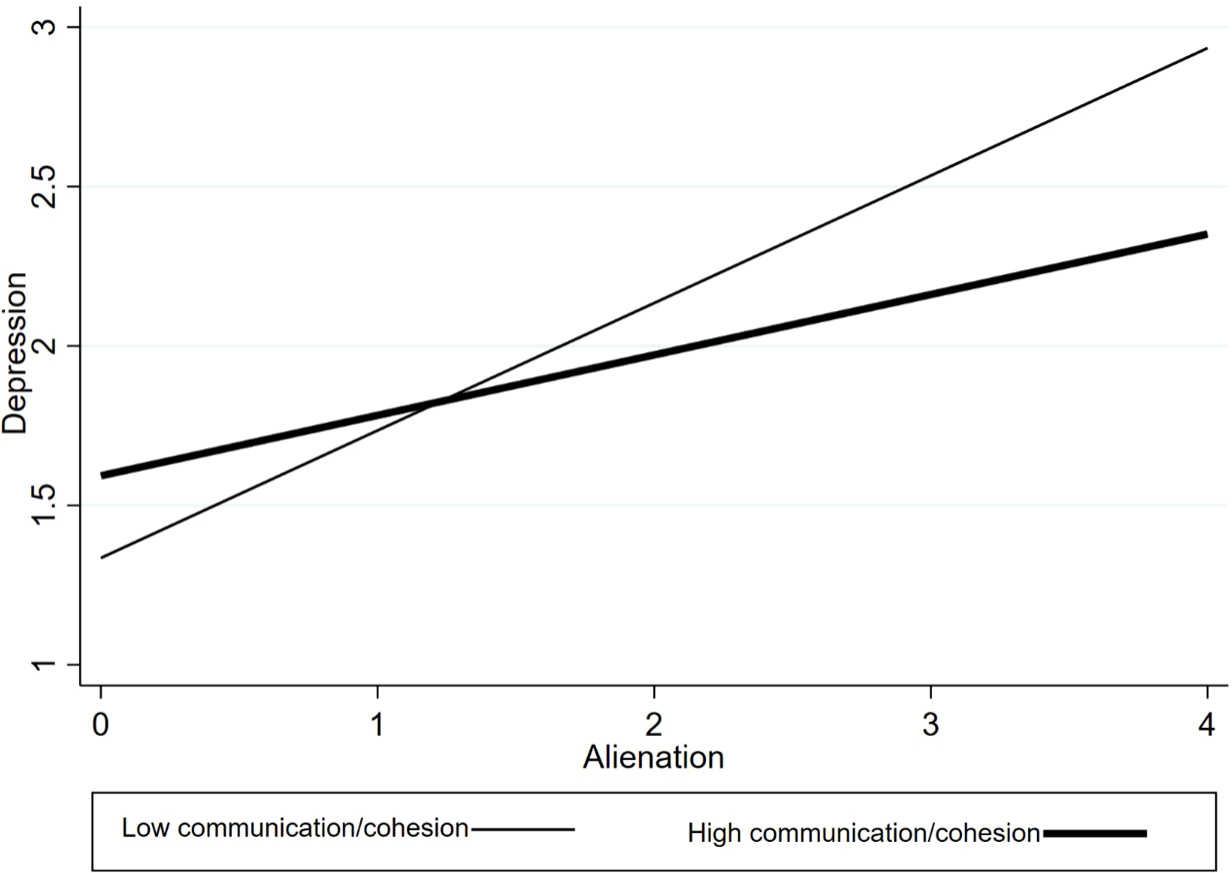
Interaction between alienation and perceived social support in predicting depression.

**Figure 3:**
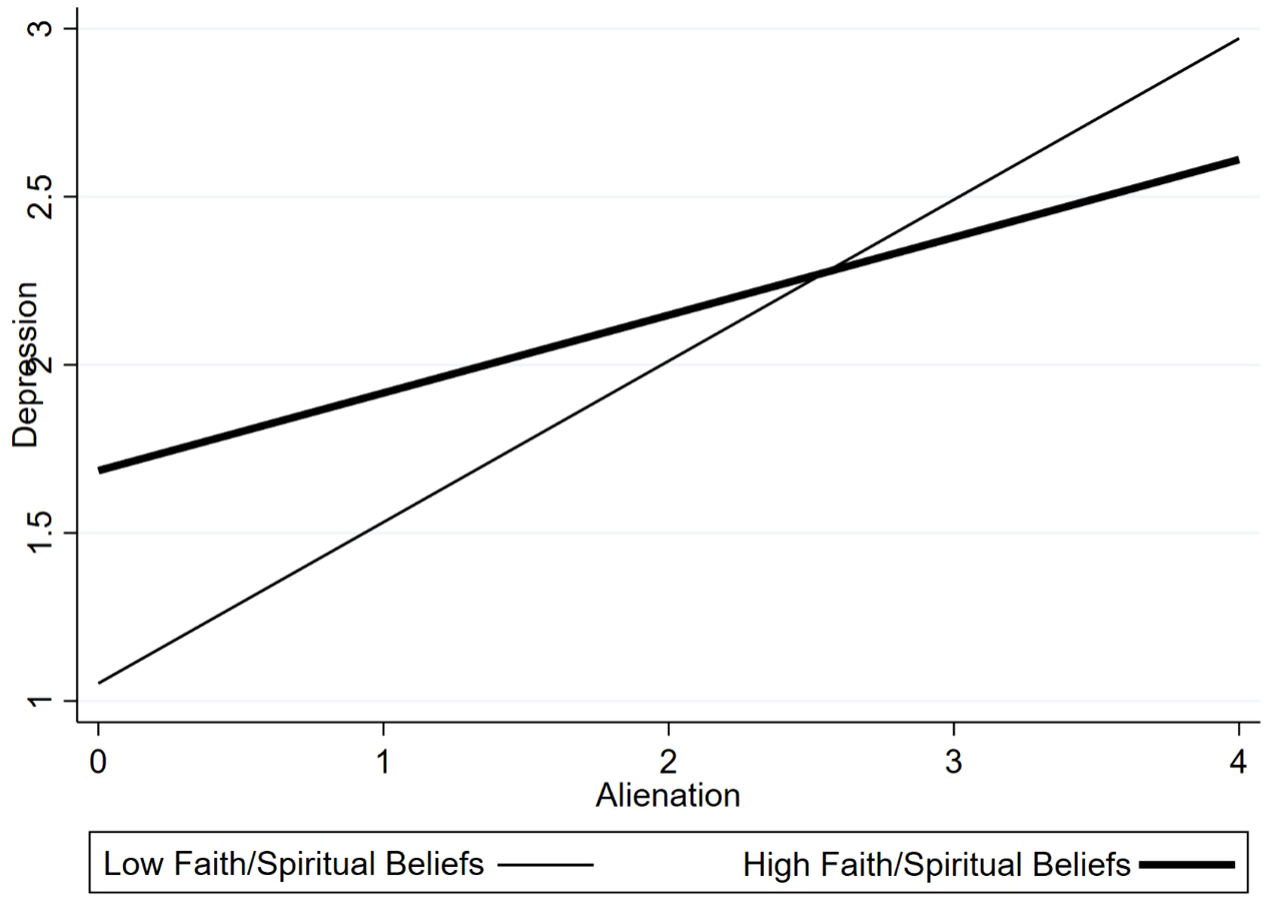
Interaction between alienation and faith/spiritual beliefs in predicting depression.

For significant interactions, simple slope analyses were conducted to examine the association between alienation and depression at high and low levels of the moderator (± 1 SD from the mean). Significant interaction effects were also plotted for visualization (see **Figures 1**–**3**), and simple slope statistics are reported in the Results section.

All analyses were performed using Stata 17.0. A two-tailed p < 0.05 was considered statistically significant. Multicollinearity diagnostics indicated no problematic collinearity among predictors (tolerance values > 0.70; variance inflation factors < 1.5). Regression assumptions were evaluated through inspection of residual-versus-fitted plots (linearity and homoscedasticity) and normal probability plots of standardized residuals, with no substantial deviations observed.

## 3 Results

### 3.1 Descriptive findings

Descriptive statistics for the study of variables and covariates are presented in **Table 2**.

**Table 2:** Descriptive statistics of study variables and covariates (N = 205).

Continuous variables						
Variable	Min	Max	M	SD	Skewness	Kurtosis
Age	25	76	44.688	11.647	.844	2.840
Depression (CES-D mean score)	1.000	3.905	2.129	.859	.286	1.759
Alienation	1.067	3.733	2.411	.715	-.019	2.038
Family resilience (Communication/Cohesion)	1.000	4.000	2.628	.724	-.331	2.463
Family resilience (Social Support)	1.000	4.000	2.750	.672	-.574	2.769
Family resilience (Family Coping Ability)	1.000	4.000	2.697	.658	-.498	3.290
Family resilience (Faith/Spirituality)	1.000	4.000	1.013	.988	.565	2.482
Categorical variables						
Variable	Category			N (%)		
Gender	Female			165 (80.5%)		
	Male			40 (19.5%)		
Education	Junior high school or below			100 (48.8%)		
	High school or above			105 (51.2%)		
Chronic illness	Yes			88 (42.9%)		
	No			117 (57.1%)		

Continuous variables are reported as mean (SD). Categorical variables are reported as frequency and percentage. Skewness and kurtosis are reported for continuous variables to assess distributional properties relevant to regression assumptions.

Group differences. Several sociodemographic differences emerged in depressive symptoms. Female caregivers reported significantly higher levels of depressive symptoms than male caregivers (*p* < 0.05; see **Table 3**). Caregivers with lower educational attainment reported higher levels of depression than those with higher education (*p* < 0.01), and caregivers with chronic illness also reported higher depressive symptoms than those without chronic illness (*p* < 0.05). No significant differences in depressive symptoms were observed across age groups.

With respect to alienation, caregivers with lower education levels and those with chronic illness reported significantly higher alienation scores (both *p* < 0.05; see **Table 3**). No significant gender or age differences were observed for alienation.

**Table 3:** Group differences in alienation and depression by caregiver characteristics (N = 205).

Variable	Group	*N*	Alienation			Depression		
			M	SE	t/F	M	SE	t/F
Gender	Male	40	2.129	.124	–.641	2.172	.111	–2.007*
	Female	165	2.217	.060		2.416	.049	
Age group	20s	5	2.057	.801	1.27	2.480	.511	.97
	30s	79	2.152	.790		2.361	.667	
	40s	59	2.350	.768		2.467	.626	
	50s	34	2.010	.770		2.198	.639	
	≥ 60	28	2.272	.694		2.398	.712	
Education	Middle school or below	100	2.330	.074	2.406*	2.496	.068	2.760**
	High school or above	105	2.075	.076		2.247	.059	
Chronic illness	No	117	2.064	.073	-2.992**	2.268	.058	–2.555*
	Yes	88	2.379	.076		2.502	.071	

Alienation = mean score on the generalized alienation scale; depression = mean CES-D score. Gender: 0 = male, 1 = female. Education level: 0 = junior middle school or below, 1 = senior high school or above. Chronic illness status: 0 = no chronic illness, 1 = at least one chronic health condition. Values are reported as means with standard errors (SE). p < 0.05*, p < 0.01**.

For family resilience, patterns varied across subdimensions and sociodemographic characteristics (see **Table 4**). With respect to gender, male caregivers reported significantly higher perceived family coping ability than female caregivers (*p* < 0.01), whereas no significant gender differences were observed for communication/cohesion, perceived social support, or faith/spiritual beliefs.

Regarding educational attainment, caregivers with lower education levels reported significantly higher scores on the faith/spiritual beliefs dimension (*p* < 0.001), while no significant educational differences were found for communication/cohesion, perceived social support, or family coping ability.

In terms of health status, caregivers without chronic illness reported significantly higher perceived family coping ability than those with chronic illness (*p* < 0.05). No significant differences by chronic illness status were observed for communication/cohesion, perceived social support, or faith/spiritual beliefs.

**Table 4:** Group differences in family resilience subscales by caregiver characteristics (N = 205).

Variable	Group	*N*	Communication/Cohesion			Social support			Family coping ability			Faith/Spirituality		
			M	SE	t/F	M	SE	t/F	M	SE	t/F	M	SE	t/F
Gender	Male	40	2.763	.145	1.573	2.871	.125	1.750	2.925	.098	2.709**	1.381	.080	–.216
	Female	165	2.512	.066		2.626	.063		2.612	.061		1.402	.049	
Age group	20s	5	2.1	.778	1.21	2.567	.401	.82	2.933	.279	.73	1.4	.652	.83
	30s	79	2.643	.856		2.673	.761		2.65	.718		1.307	.492	
	40s	59	2.415	.838		2.573	.801		2.571	.849		1.492	.642	
	50s	34	2.706	.84		2.882	.858		2.765	.815		1.417	.743	
	≥ 60	28	2.542	.955		2.655	.923		2.798	.699		1.429	.623	
Education	Middle school or below	100	2.493	.087	–1.095	2.585	.086	–1.543	2.597	.081	–1.398	1.565	.069	3.978***
	High school or above	105	2.625	.083		2.759	.072		2.746	.069		1.238	.045	
Chronic illness	No	117	2.554	.082	–.132	2.668	.073	–.12	2.755	.074	1.812*	1.397	.055	–.003
	Yes	88	2.57	.089		2.682	.088		2.564	.075		1.398	.065	

Subscales: Communication/Cohesion, Social Support, Family Coping Ability, Faith/Spirituality. p <.05*, p <.01**, p <.001**

Correlations. **Table 5** presents the zero-order correlations. Alienation was strongly positively correlated with depression (r = 0.58, p < 0.001). The resilience subdimensions were moderately negatively correlated with both alienation and depression (rs ≈ –0.30 to –0.50) and moderately intercorrelated (rs ≈ 0.30–0.60). No correlation exceeded 0.70, indicating an absence of problematic multicollinearity.

**Table 5:** Correlation matrix for study variables (N = 205).

Variable	1	2	3	4	5	6	7
Gender	1						
Age	-.179*	1					
Education level	-.111	-.131	1				
Chronic illness status	-.095	.035	-.100	1			
Alienation	.148	-.013	-.191*	-.178*	1		
Depression	.046	.014	-.167**	-.203**	.623***	1	
Family resilience	-.140	.033	.103	-.018	-.437***	-.314***	1

Gender (0 = male, 1 = female); age (years); education level (0 = junior middle school or below, 1 = senior high school or above); chronic illness status (0 = no chronic illness, 1 = at least one chronic health condition). The overall family resilience score was included for descriptive correlation purposes only and was not entered into the regression or moderation analyses, which focused exclusively on the resilience subdimensions. p < 0.05*, p < 0.01**, p < 0.001***.

Depression and alienation. Caregivers showed considerable variation in depressive symptoms. The mean CES-D item score was 2.13 (SD = 0.86), with higher values indicating greater depressive symptoms. Alienation levels were also relatively elevated to 2.41 (SD = 0.72), suggesting frequent experiences of social withdrawal or misunderstanding.

Family resilience. Across the four family resilience dimensions, mean scores were 2.63 for communication/cohesion, 2.75 for perceived social support, 2.70 for family coping ability, and 1.01 for faith/spiritual beliefs. Overall, caregivers reported moderate levels of family functioning across most domains, whereas faith/spiritual beliefs were notably lower on average. The subsequent results are summarized in **Tables 3**–**5**. The corresponding analyses are reported in **Table 3**, with additional results presented in **Tables 4**, **5**.

### 3.2 Alienation as a predictor of caregiver depression

Alienation as a predictor of caregiver depression. **Table 6** presents hierarchical regression models predicting caregiver depressive symptoms. In Model 1, which included only the control variables, the model explained a modest but statistically significant proportion of variance in depression (F (4, 200) = 3.47, *p* < 0.01, R² = 0.07). Caregivers with lower educational attainment and those with chronic illness reported higher levels of depressive symptoms, whereas gender showed a marginal association and age was not statistically significant.

**Table 6:** Hierarchical regression of caregiver depression on alienation and covariates (N = 205).

Predictor	Model 1			Model 2		
	B (SE)	β	t	B (SE)	β	t
Gender (female)	.092 (.137)	.047	.67	–.078 (.111)	–.040	–.70
Age	–.000 (.005)	–.004	.05	.000 (.004)	.006	.10
Education (high vs. low)	–.219 (.108)	–.142*	–2.03	–.071 (.088)	–.046	–.80
Chronic illness (no vs. yes)	.300 (.107)	.193**	2.80	.135 (.088)	.087	1.54
Alienation				.712 (.067)	.605***	10.56
R²	.065			.400		
Adj. R²	.046			.386		
ΔR²				.340***		
F	3.47***			26.61***		

Male (gender), junior middle school or below (education), and no chronic illness (health status). Age was entered as a continuous variable. p <.05*, p <.01**, p <.001***.

In Model 2, the inclusion of alienation significantly improved model fit. Alienation was positively and significantly associated with depressive symptoms (*p* < 0.001), and the explained variance increased substantially relative to Model 1 (ΔR², *p* < 0.001), indicating that higher alienation was associated with higher levels of caregiver depression after adjusting for sociodemographic covariates.

### 3.3 Moderating effect of family resilience subdimensions

Moderation by family resilience subdimensions. Separate moderated regression models were estimated for each family resilience subdimension (**Table 7**). Across all models, alienation remained significantly associated with depressive symptoms. Significant interaction effects were observed for three subdimensions—communication/cohesion, perceived social support, and faith/spiritual beliefs—indicating that these family processes moderated the association between alienation and depression. In contrast, the interaction between alienation and perceived family coping ability was not statistically significant.

**Table 7:** Moderated regression of the alienation–depression association by family resilience subdimensions (Model 3, N = 205).

Predictor	Communication & cohesion			Social support			Family coping ability			Faith/Spiritual beliefs		
	B (SE)	β	t	B (SE)	β	t	B (SE)	β	t	B (SE)	β	t
Gender (female)	–.045 (.108)	–.023	–.42	.061 (.112)	.031	.55	–.079 (.112)	–.041	–.71	–.075 (.109)	–.039	–.69
Age	.000 (0.005)	.009	.09	.000 (.004)	–.001	–.02	.000 (.004)	–.002	–.02	.000 (.004)	–.001	–.02
Education (high vs. low)	–.077 (0.105)	–.048	–.73	–.057 (.087)	–.037	–.65	–.057 (.087)	–.035	–.60	–.057 (.087)	–.035	–.60
Chronic illness (no vs. yes)	.111 (.086)	.071	1.29	.112 (.087)	.072	1.28	.128 (.092)	.096	1.39	.150 (.096)	.109	1.73
Alienation	.661 (.072)	.561***	9.14	.692 (.072)	.588***	9.65	.672 (.071)	.572***	9.48	.705 (.067)	.599***	10.60
Communication & Cohesion	–.079 (.053)	–.088	–1.47									
Social Support				–.043 (.057)	–.045	–.77						
Family Coping Ability							–.089 (.060)	–.088	–1.49			
Faith/Spiritual Beliefs										.023 (.073)	.018	.32
Communication × Alienation	–.254 (.068)	–.204***	–3.72									
Social Support × Alienation				–.172 (.072)	–.133*	–2.40						
Family Coping × Alienation							–.115 (.074)	–.086	–1.55			
Faith × Alienation										–.365 (.128)	–.156**	–2.84
R²	.443			.419			.414			.424		
Adj. R²	.423			.398			.383			.404		
F	22.33***			20.27***			19.87***			20.74***		

Each column represents a separate regression model including covariates (gender, age, education, chronic illness), alienation, one family resilience subdimension, and the corresponding interaction term. Reference categories are male (gender), junior middle school or below (education), and not having a chronic illness (health status). Age was entered as a continuous variable. *p <.05, **p <.01, ***p <.001.

### 3.4 Resilience subdimensions

#### 3.4.1 Communication and cohesion

The interaction between alienation and family communication/cohesion was statistically significant (β =–0.204, p <0.001). Simple slope analyses (**Figure 1**) indicated that the association between alienation and depressive symptoms was stronger among caregivers reporting lower levels of family cohesion (p < 0.001), whereas this association was weaker among those with higher cohesion (p< 0.05). These findings indicate that family communication and emotional cohesion moderate the association between alienation and caregiver depression.

#### 3.4.2 Perceived social support

The interaction between alienation and perceived social support was statistically significant (β =–0.133, p <0.05). Simple slope analyses (**Figure 2**) indicated that the association between alienation and depressive symptoms was stronger at lower levels of perceived social support (p < 0.001), whereas this association was attenuated, though still positive, at higher levels of support (p= 0.01). These findings indicate that perceived social support moderates the association between alienation and depressive symptoms.

#### 3.4.3 Perceived family coping ability

The interaction between alienation and perceived family coping ability was not statistically significant (β =–0.086, p = 0.22). Alienation remained significantly associated with depressive symptoms, whereas the interaction term did not reach statistical significance. These results indicate that perceived family coping ability did not moderate the association between alienation and caregiver depression.

#### 3.4.4 Faith/spiritual beliefs

The interaction between alienation and faith/spiritual beliefs was statistically significant (β =–0.156, p <0.01). Simple slope analyses (**Figure 3**) showed that among caregivers reporting higher levels of faith/spiritual beliefs, the association between alienation and depressive symptoms was weak and not statistically significant (p > 0.10). In contrast, among caregivers with lower levels of faith/spiritual beliefs, alienation was significantly associated with higher depressive symptoms (p< 0.001). These findings indicate that faith/spiritual beliefs moderate the association between alienation and caregiver depression.

### 3.5 Summary

Across the four moderation models, three family resilience subdimensions—communication/cohesion, perceived social support, and faith/spiritual beliefs—significantly moderated the association between alienation and depressive symptoms. In contrast, perceived family coping ability did not show a significant moderating effect. Across all models, alienation remained significantly associated with caregiver depressive symptoms. The effects of the control variables were consistent with earlier models, with lower education and chronic illness associated with higher depressive symptoms, gender showing a marginal association, and age not significantly related to depression.

## 4 Discussion

This study examined how alienation relates to depressive symptoms among Chinese caregivers of children with ASD and whether specific components of family resilience mitigate this association. Consistent with the study hypotheses, alienation was strongly associated with higher levels of depressive symptoms, and three family resilience subdimensions—communication/cohesion, perceived social support, and faith/spiritual beliefs—demonstrated significant buffering effects. Together, these findings highlight alienation as a salient psychosocial risk factor associated with caregiver depression and underscore the importance of distinct family processes in shaping caregivers’ psychological adjustment within the present cultural context.

Consistent with prior research on stigma, social exclusion, and caregiver well-being, caregivers who reported higher levels of alienation also reported substantially higher depressive symptoms (24, 25, 38). Alienation reflects experiences of being misunderstood, marginalized, or socially excluded, and the internalization of such experiences may undermine emotional well-being by reducing perceived belonging and support. In the Chinese context, these associations may be shaped by uneven public understanding of ASD and by the social expectations placed on families, which can intensify caregivers’ sense of isolation while they manage demanding caregiving responsibilities. The present findings suggest that alienation is not merely a secondary burden but a central correlation of caregivers’ depressive symptoms.

A key contribution of this study lies in identifying specific dimensions of family resilience that moderate the association between alienation and depression. In line with our analytic focus, moderation effects were examined for each resilience subdimension rather than for an overall composite. Communication and cohesion showed the strongest buffering effect, indicating that families characterized by open communication and emotional connectedness may provide caregivers with supportive environments in which concerns can be shared and validated, thereby reducing the emotional impact of alienation (27, 39). Perceived social support similarly attenuated the alienation–depression association, suggesting that connections with extended family, peers, and community networks may compensate for feelings of social disconnection. Faith or spiritual beliefs constituted a third buffering dimension; shared belief systems and meaning-making processes may help caregivers reinterpret marginalizing experiences and sustain emotional equilibrium in the context of elevated alienation. These patterns are consistent with intervention- and practice-oriented literature suggesting that strengthening caregiver/family resilience and enhancing accessible support networks may improve caregiver adjustment and well-being (40–44).

In contrast, perceived family coping ability did not significantly moderate the relationship between alienation and depression. Coping efficacy may be more closely related to managing practical caregiving challenges than to buffering the relational and emotional dimensions of alienation. Additionally, the relatively low coping scores observed in this sample may have limited variability, reducing the likelihood of detecting interaction effects. These findings underscore that family resilience is multidimensional and that relational and meaning-oriented processes may be particularly relevant for caregivers’ emotional well-being.

### 4.1 Practical implications

The findings have several practical implications for practice and policy. Interventions aimed at reducing caregiver depression may benefit from explicitly addressing alienation through stigma-reduction efforts, inclusive community initiatives, and peer support programs that foster a sense of belonging. At the family level, strengthening communication, cohesion, and social support networks may help mitigate the psychological consequences of alienation. Meaning-focused or culturally grounded approaches may be especially relevant for caregivers who draw on spiritual or belief-based frameworks for support. Consistent with the strong association between alienation and depressive symptoms, addressing caregiver alienation represents a potentially important intervention target. Community-based initiatives may benefit from actively challenging stigma, enhancing public understanding of ASD, and promoting social inclusion of families of children with ASD (5, 45). Public education efforts and non-stigmatizing practices within health and social services may help reduce blaming attitudes and foster more supportive community environments.

Second, building peer support networks appears to be an important strategy. Whether delivered in person or online, peer groups can provide validation, shared experiences, and practical guidance, thereby counteracting feelings of isolation. Incorporating structured peer support into caregiver mental health programs may be particularly beneficial, as lower levels of alienation are associated with fewer depressive symptoms.

Third, strengthening family resilience offers a meaningful pathway for supporting caregiver mental health. Interventions may target the specific resilience components identified in this study. Programs that enhance communication and cohesion—by facilitating open emotional expression, collaborative problem-solving, and mutual support—may be especially impactful. Family-based psychoeducation or therapeutic approaches can help reinforce these relational processes while supporting families in managing stress.

Fourth, enhancing broader social support and service navigation represents another important direction (46). Practitioners can connect families to community resources, parent-mentor networks, and supportive organizations, and can assist caregivers in developing help-seeking and social networking skills. For caregivers who already draw on spiritual or meaning-focused frameworks, interventions that cultivate shared beliefs, purpose, or meaning may help sustain optimism and reduce vulnerability to alienation. Importantly, such approaches should be culturally sensitive and aligned with families’ existing values; even in non-religious families, encouraging shared narratives of growth, solidarity, or collective strength may serve similar supportive functions.

Given that communication/cohesion, perceived social support, and faith/spiritual beliefs emerged as the most salient moderators in this study, interventions that prioritize emotional and relational strengthening may be more effective for addressing depressive symptoms than approaches focused solely on practical coping skills. Ideally, comprehensive programs should integrate relational support with problem-focused strategies to provide holistic assistance for caregivers of children with ASD.

Importantly, these implications are equity-oriented: accessible, low-threshold community and family supports (e.g., anti-stigma initiatives, peer networks, and family-centered guidance) may reduce psychological burdens without requiring high-cost resources or specialized services. Prioritizing inclusive and culturally responsive support opportunities for under-served caregiver groups may help address inequities in caregiver mental health linked to differential access to services and social participation.

### 4.2 Limitations and future research

This study has several limitations. First, the cross-sectional design precludes causal inference. Although our framework posits that alienation contributes to depression and that family resilience buffers this association, the relationships may be reciprocal (e.g., depressive symptoms may increase social withdrawal and undermine family communication or perceived support). Longitudinal designs are therefore needed to establish temporal ordering and to test potential bidirectional dynamics among alienation, family resilience, and depression.

Second, all measures relied on caregiver self-report, which may introduce common method variance and response biases. Future work should strengthen measurement validity using multi-informant data and complementary assessment modalities (e.g., clinical evaluation of depression, observational indicators of family interaction, or objective indices of social connectedness).

Third, the sample was drawn from a single province in Northeast China and was recruited largely through community organizations and service agencies, which may limit generalizability and may overrepresent families already connected to support resources. In addition, although the FaRE demonstrated strong psychometric performance after item reduction, it was originally developed in the context of physical illness and may not fully capture autism-specific resilience processes. Replication in more diverse settings and with ASD-tailored resilience measures is warranted.

Building on these limitations, future research should extend our findings in several directions. Methodologically, longitudinal and mixed-method designs (including qualitative interviews or social network approaches) could provide a more nuanced account of caregivers’ lived experiences of alienation and support and clarify how resilience processes develop over time. Analytically, moderation could be probed more precisely using the Johnson–Neyman technique, which identifies the range(s) of the moderator for which the focal association is statistically significant (i.e., the region of significance) and complements conventional simple-slope probing (e.g., at ±1 SD). Theoretically, additional factors—particularly child characteristics such as ASD symptom severity, functional level, and challenging behaviors—should be examined as potential contributors to caregiver alienation and depression and as potential contingencies in resilience processes (47, 48). Finally, it may be useful to further differentiate alienation into interpersonal, societal, and self-related components to identify which facets are most strongly linked to depressive symptoms.

Importantly, intervention implications derived from this work require empirical evaluation. Controlled trials are needed to test whether multi-component programs that reduce caregiver alienation (e.g., stigma-reduction, inclusive community practices, peer support) and strengthen key resilience processes (communication/cohesion, social support, and meaning-focused coping) can reduce caregiver depression beyond standard care.

## 5 Conclusion

This study highlights the psychosocial challenges faced by caregivers of children with ASD and identifies both risk processes and moderating resilience mechanisms associated with their mental health. Alienation—characterized by experiences of social disconnection, marginalization, and stigma—was strongly associated with depressive symptoms, underscoring the importance of considering the broader social context surrounding autism in addition to the clinical needs of children.

The findings also suggest potentially protective processes within families. Although not all aspects of family resilience operated similarly, three subdimensions—communication/cohesion, perceived social support, and faith/spiritual beliefs—demonstrated significant buffering effects on the association between alienation and depression. Families characterized by open communication, emotional closeness, supportive networks, and shared belief systems appeared to provide environments that helped caregivers manage the psychological burden of social exclusion.

Taken together, the results point to two complementary avenues for supporting caregiver well-being. Reducing external stressors—such as stigma, misinformation about ASD, and limited community inclusion—may help alleviate feelings of alienation. Strengthening internal family resources, particularly relational and meaning-centered processes, may further support caregivers in coping with the challenges of raising a child with ASD. Interventions may benefit from integrating both approaches by addressing societal attitudes while also enhancing family-level resilience capacities.

Ultimately, caregiver depression in the context of ASD is not only an individual mental health concern but also a social one. This study provides evidence of a strong association between alienation and depressive symptoms, while highlighting specific family resilience processes that function as meaningful buffers. Supporting caregivers in feeling more connected—to their families, communities, and sources of meaning—remains an important goal for promoting sustained well-being among caregivers of children with ASD.

## Data Availability

The raw data supporting the conclusions of this article will be made available by the authors, without undue reservation.

## References

[1] Maenner MJ Warren Z Williams AR Amoakohene E Bakian AV Bilder DA . Prevalence and characteristics of autism spectrum disorder among children aged 8 years — Autism and developmental disabilities monitoring network, 11 sites, United States 2020. MMWR Surveill Summ. (2023) 72:1–14. doi: 10.15585/mmwr.ss7202a1, PMID: 36952288 PMC10042614

[2] Cubells JF . Prevalence of autism spectrum disorders in China. Shanghai Arch Psychiatry. (2013) 25:176–8. doi: 10.3969/j.issn.1002-0829.2013.03.008, PMID: 24991154 PMC4054547

[3] Dai Q Xu X Wang H Zhao Z Zhang X Zhou A . Prevalence of autism spectrum disorder in Chinese children from 2010 to 2016: A meta-analysis. Chin J Child Health Care. (2017) 25:1243–8.

[4] American Psychological Association . Publication manual of the american psychological association7th ed. Washington, DC: American Psychological Association (2020).

[5] Ng CSM Ng SSL . A qualitative study on the experience of stigma for Chinese parents of children with autism spectrum disorder. Sci Rep. (2022) 12:19550. doi: 10.1038/s41598-022-23978-0, PMID: 36379973 PMC9666461

[6] Zhang P Wang M Feng S . Identifying research hotspots in mental health for parents of children with autism spectrum disorder: A bibliometric perspective. Humanities Soc Sci Commun. (2025) 12:1881. doi: 10.1057/s41599-025-06152-5, PMID: 39310270

[7] Zheng L Long C Choi W . The effect of social activities on the alienation and family resilience of Chinese caregivers for children with autism: A latent class analysis. Front Psychiatry. (2024) 15:1406073. doi: 10.3389/fpsyt.2024.1406073, PMID: 38895029 PMC11184502

[8] Li F Tang Y Li F Fang S Liu X Tao M . Psychological distress in parents of children with autism spectrum disorder: A cross-sectional study based on 683 mother–father dyads. J Pediatr Nurs. (2022) 65:e49–55. doi: 10.1016/j.pedn.2022.02.006, PMID: 35249769

[9] Alibekova R Chan CK Crape B Kadyrzhanuly K Gusmanov A An S . Stress, anxiety, and depression in parents of children with autism spectrum disorders in Kazakhstan: Prevalence and associated factors. Global Ment Health. (2022) 9:472–82. doi: 10.1017/gmh.2022.51, PMID: 36618742 PMC9806964

[10] Wang C . Mental health and social support of caregivers of children and adolescents with ASD and other developmental disorders during the COVID-19 pandemic. J Affect Disord Rep. (2021) 6:100242. doi: 10.1016/j.jadr.2021.100242, PMID: 34580666 PMC8457638

[11] Kostiukow A Poniewierski P Janowska D Samborski W . Levels of happiness and depression in parents of children with autism spectrum disorder in Poland. Acta Neurobiologiae Experimentalis. (2021) 81:279–85. doi: 10.21307/ane-2021-026, PMID: 34672298

[12] Eshraghi AA Cavalcante L Furar E Alessandri M Eshraghi RS Armstrong FD . Implications of parental stress on worsening of behavioral problems in children with autism during COVID-19 pandemic: “the spillover hypothesis. Mol Psychiatry. (2022) 27:1869–70. doi: 10.1038/s41380-021-01433-2, PMID: 35064235 PMC8780050

[13] Lievore R Lanfranchi S Mammarella IC . Parenting stress in autism: Do children’s characteristics still count more than stressors related to the COVID-19 pandemic? Curr Psychol. (2024) 43:2607–17. doi: 10.1007/s12144-023-04441-3, PMID: 37359637 PMC10014138

[14] Kerns CM Rast JE Shattuck PT . Prevalence and correlates of caregiver-reported mental health conditions in youth with autism spectrum disorder in the United States. J Clin Psychiatry. (2020) 82:11637. doi: 10.4088/JCP.20m13242, PMID: 33356021

[15] Sartor T Sons S Kuhn JT Tröster H . Coping resources and stress due to demands in parents of children with autism spectrum disorder. Front Rehabil Sci. (2023) 4:1240977. doi: 10.3389/fresc.2023.1240977, PMID: 37869574 PMC10588644

[16] Chen X Tong J Zhang W Wang X Ma S Shi D . Factors predicting depressive symptoms in parents of children with autism spectrum disorder in eastern China. BMC Public Health. (2024) 24:226. doi: 10.1186/s12889-024-17731-7, PMID: 38238720 PMC10797873

[17] Goffman E . Stigma: Notes on the management of spoiled identity. New York, NY: Simon & Schuster (2009).

[18] Ng CK Lam SH Tsang ST Yuen CM Chien CW . The relationship between affiliate stigma in parents of children with autism spectrum disorder and their children’s activity participation. Int J Environ Res Public Health. (2020) 17:1799. doi: 10.3390/ijerph17051799, PMID: 32164271 PMC7084220

[19] Du X Su X Ding D Zhu Y Sun Y Wang M . Relationship between family functioning and affiliate stigma in parents of children with autism spectrum disorder in China: The mediating role of positive aspects of caregiving. Front Psychiatry. (2025) 16:1613340. doi: 10.3389/fpsyt.2025.1613340, PMID: 40761600 PMC12320499

[20] Chen H Ding Y Xu D Xiong Z . Resilience and affiliate stigma among parents of children with autism spectrum disorder: The mediating role of life satisfaction and the moderating role of ostracism. Psychol Res Behav Manage. (2025) 18:1519–29. doi: 10.2147/PRBM.S524580, PMID: 40607138 PMC12219156

[21] Ye Y Deng T Chen M Huang B Ji Y Feng Y . Relationship between affiliate stigma and family quality of life among parents of children with autism spectrum disorders: The mediating role of parenting self-efficacy. Arch Psychiatr Nurs. (2024) 49:23–31. doi: 10.1016/j.apnu.2024.01.011, PMID: 38734451

[22] Salami S Alhalal E . Affiliate stigma among caregivers of children with autism spectrum disorder: The role of coping strategies and perceived social support. J Disability Res. (2024) 3:20240009. doi: 10.57197/JDR-2024-0009

[23] Zhou T Wang Y Yi C . Affiliate stigma and depression in caregivers of children with autism spectrum disorders in China: Effects of self-esteem, shame and family functioning. Psychiatry Res. (2018) 264:260–5. doi: 10.1016/j.psychres.2018.03.071, PMID: 29655969

[24] Chen WC Chen SJ Zhong BL . Sense of alienation and its associations with depressive symptoms and poor sleep quality in older adults who experienced the lockdown in Wuhan, China, during the COVID-19 pandemic. J Geriatric Psychiatry Neurol. (2022) 35:215–22. doi: 10.1177/08919887221078564, PMID: 35130783 PMC8899829

[25] Salleh NS Tang LY Husain M Abdullah KL Kueh YC . Affiliate stigma, resilience and quality of life among parents of children with autism spectrum disorder in two public hospitals in Kelantan, Malaysia. Malaysian J Med Sci. (2024) 31:217–26. doi: 10.21315/mjms2024.31.3.17, PMID: 38984240 PMC11229570

[26] Alicka Y Gjeta A Kotherja O Topi B Osmanaj E Shyti M . The levels of stress, anxiety, and depression in parents of children with autism spectrum disorder: A review of the literature. Multidiscip Rev. (2025) 8:2025350. doi: 10.31893/multirev.2025350

[27] Walsh F . Strengthening family resilience. New York, NY: Guilford Press (2015).

[28] Faccio F Gandini S Renzi C Fioretti C Crico C Pravettoni G . Development and validation of the family resilience (FaRE) questionnaire: An observational study in Italy. BMJ Open. (2019) 9:e024670. doi: 10.1136/bmjopen-2018-024670, PMID: 31171547 PMC6561460

[29] Pastor-Cerezuela G Fernández-Andrés MI Pérez-Molina D Tijeras-Iborra A . Parental stress and resilience in autism spectrum disorder and Down syndrome. J Family Issues. (2021) 42:3–26. doi: 10.1177/0192513X20910192, PMID: 41804314

[30] Radloff LS . The CES-D scale: A self-report depression scale for research in the general population. Appl psychol Measurement. (1977) 1:385–401. doi: 10.1177/014662167700100306, PMID: 26918431

[31] Wang M Armour C Wu Y Ren F Zhu X Yao S . Factor structure of the CES-D and measurement invariance across gender in Mainland Chinese adolescents. J Clin Psychol. (2013) 69:966–79. doi: 10.1002/jclp.21978, PMID: 23775279

[32] Yang D Zhang J Huang X . Adolescent student’s sense of alienation: theoretical construct and scale development. Acta Psychologica Sin. (2002) 34:77–83.

[33] Wang G Dong J Zhu N Zhu Y . Development and validation of a social alienation predictive model for older maintenance hemodialysis patients based on latent profile analysis—a cross-sectional study. BMC Geriatrics. (2024) 24:495–5. doi: 10.1186/S12877-024-05116-9, PMID: 38840071 PMC11154990

[34] Wang W Yang Y Song C Liu Q Mu R Yu D . Suicidal risk among Chinese parents of autistic children and its association with perceived discrimination, affiliate stigma, and social alienation. BMC Psychiatry. (2024) 24:784. doi: 10.1186/s12888-024-06252-7, PMID: 39523356 PMC11552419

[35] Li M Ma R Zhang S Wang S Jiao J Liu L . Reliability and validity of the Chinese version of the family resilience questionnaire (FaRE questionnaire) in patients with breast cancer: A cross-sectional study. BMJ Open. (2022) 12:e051093. doi: 10.1136/bmjopen-2021-051093, PMID: 35443942 PMC9021805

[36] Lin Y Wang Y Lin C Ni Q Jia R Chang Y . The mediating role of perceived social support: Alexithymia and parental burnout in parents of children with autism spectrum disorder. Front Psychol. (2023) 14:1139618. doi: 10.3389/fpsyg.2023.1139618, PMID: 37359855 PMC10290202

[37] Hayes AF . Introduction to mediation, moderation, and conditional process analysis: A regression-based approach. 2nd ed. New York, NY: Guilford Press (2017).

[38] Wang S Wu T Liu J Guan W . Relationship between perceived discrimination and social anxiety among parents of children with autism spectrum disorders in China: The mediating roles of affiliate stigma and perceived social support. Res Autism Spectr Disord. (2024) 111:102310. doi: 10.1016/j.rasd.2023.102310, PMID: 41802445

[39] Qi W Shi J Cui L . Family contagion of mental toughness and its influence on youth mental well-being: Family cohesion as a moderator. Pers Individ Dif. (2023) 202:111963. doi: 10.1016/j.paid.2022.111963, PMID: 41802445

[40] Kotera Y Pope M Chircop J Kirkman A Bennett-Viliardos LA Sharaan S . Resilience intervention for families of autistic children: Reviewing the literature. Br J Psychiatry. (2021) 194:500–9. doi: 10.54127/SWJS6679

[41] Ghanouni P Hood G . Stress, coping, and resiliency among families of individuals with autism: A systematic review. Rev J Autism Dev Disord. (2021) 8:389–402. doi: 10.1007/s40489-021-00245-y, PMID: 41804457

[42] Otis-Green S Juarez G . Enhancing the social well-being of family caregivers. Semin Oncol Nurs. (2012) 28:246–55. doi: 10.1016/j.soncn.2012.09.007, PMID: 23107182 PMC3729043

[43] Benson PR . Network characteristics, perceived social support, and psychological adjustment in mothers of children with autism spectrum disorder. J Autism Dev Disord. (2012) 42:2597–610. doi: 10.1007/s10803-012-1517-9, PMID: 22484793

[44] Bi XB He HZ Lin HY Fan XZ . Influence of social support network and perceived social support on the subjective wellbeing of mothers of children with autism spectrum disorder. Front Psychol. (2022) 13:835110. doi: 10.3389/fpsyg.2022.835110, PMID: 35401352 PMC8989138

[45] Chen X Tong J Jiang B Ma S Wang X Sun X . Courtesy stigma among primary caregivers of children with autism spectrum disorder in eastern China. Front Psychiatry. (2023) 14:1236025. doi: 10.3389/fpsyt.2023.1236025, PMID: 38045614 PMC10690950

[46] Ban Y Sun J Liu J . Social support and subjective well-being in Chinese parents of children with autism spectrum disorder: The mediating role of perceived discrimination. Front Psychol. (2021) 12:781794. doi: 10.3389/fpsyg.2021.781794, PMID: 34819903 PMC8606395

[47] Clauser P Ding Y Chen EC Cho SJ Wang C Hwang J . Parenting styles, parenting stress, and behavioral outcomes in children with autism. School Psychol Int. (2021) 42:33–56. doi: 10.1177/0143034320971675, PMID: 41804314

[48] Williams KL Kirby AV Watson LR Sideris J Bulluck J Baranek GT . Sensory features as predictors of adaptive behaviors: A comparative longitudinal study of children with autism spectrum disorder and other developmental disabilities. Res Dev Disabil. (2018) 81:103–12. doi: 10.1016/j.ridd.2018.07.002, PMID: 30060977 PMC7473611

